# Mesenchymal Stromal Cell Therapy in Spinal Cord Injury: Mechanisms and Prospects

**DOI:** 10.3389/fncel.2022.862673

**Published:** 2022-06-03

**Authors:** Ji-Le Xie, Xing-Ran Wang, Mei-Mei Li, Zi-Han Tao, Wen-Wen Teng, Saijilafu

**Affiliations:** ^1^Department of Orthopaedics, The First Affiliated Hospital, Soochow University, Suzhou, China; ^2^Orthopaedic Institute, School of Medicine, Soochow University, Suzhou, China

**Keywords:** mesenchymal stromal cell, cell transplanting, cell differentiation, neuroregeneration, spinal cord injury

## Abstract

Spinal cord injury (SCI) often leads to severe motor, sensory, and autonomic dysfunction in patients and imposes a huge economic cost to individuals and society. Due to its complicated pathophysiological mechanism, there is not yet an optimal treatment available for SCI. Mesenchymal stromal cells (MSCs) are promising candidate transplant cells for use in SCI treatment. The multipotency of MSCs, as well as their rich trophic and immunomodulatory abilities through paracrine signaling, are expected to play an important role in neural repair. At the same time, the simplicity of MSCs isolation and culture and the bypassing of ethical barriers to stem cell transplantation make them more attractive. However, the MSCs concept has evolved in a specific research context to encompass different populations of cells with a variety of biological characteristics, and failure to understand this can undermine the quality of research in the field. Here, we review the development of the concept of MSCs in order to clarify misconceptions and discuss the controversy in MSCs neural differentiation. We also summarize a potential role of MSCs in SCI treatment, including their migration and trophic and immunomodulatory effects, and their ability to relieve neuropathic pain, and we also highlight directions for future research.

## Introduction

Approximately 27 million people worldwide live with disability due to spinal cord injury (SCI), and approximately 0.92 million new patients are reported each year (James et al., 2019). SCI often results in partial or complete loss of sensory and motor functions in patients, resulting in huge physical and social consequences and a heavy medical burden for both patients’ families and society. The difficulty of treating SCI derives from its complex pathophysiology. First, adult neurons are terminally differentiated cells that cannot divide, and the number and distribution of endogenous neural stem cells in the spinal cord are very limited. Second, primary injury in the spinal cord often triggers a cascade of secondary damage, including the release of excitatory amino acids, loss of ionic homeostasis, cellular calcium overload, mitochondrial dysfunction, and multiple immune and inflammatory responses, which further aggravate tissue ischemia and inflammation, and lead to a cycle of neuronal and glial apoptosis. Thus, the secondary damage often exceeds the primary injury. Finally, glial scars and cystic cavities develop during the later stages of SCI and act as a physical barrier to axon regeneration (Ahuja et al., 2017a; Badhiwala et al., 2018). Thus, SCI often results in permanent neurological functional deficits. Regarding the low survival of patients and the huge consumption of medical resources, the interest in developing new treatments for SCI continues. However, achievements have been limited. In fact, according to the clinical guidelines, the only drug currently available for SCI treatment is methylprednisolone, along with strict timing requirements, unclear efficacy, and high risk of complications (Fehlings et al., 2017a,b). Another drug, ganglioside GM1, was once thought to be effective, but has been withdrawn from clinical practice amid great controversy (Hurlbert et al., 2013; Walters et al., 2013).

Stem cell therapy has broadened the field of SCI research because of its potential to protect, rescue, or replace damaged nerve cells. Many types of stem cells, including embryonic stem cells (ESCs), neural precursor/stem cells (NPCs/NSCs), olfactory ensheathing cells (OECs), Schwann cells (SCs), mesenchymal stromal cells (MSCs), and induced pluripotent stem cells (iPSCs), have been investigated for the treatment of SCI (Vismara et al., 2017). Therapies using these cells have achieved promising results on the bench. However, when it comes to clinical applications, especially for ESCs and NSCs, the cell source problem, ethical dilemmas, and capricious changes in policy are unavoidable problems (Murugan, 2009; Trawczynski et al., 2019). MSCs have been considered somatic stem cells. They are easily accessible and have strong self-renewal ability and multidirectional differentiation potential, which make them attractive candidates for cell therapy for SCI treatment. MSCs can be obtained from a variety of sources for autologous transplantation, avoiding the immune and ethical issues associated with ESCs. In addition, MSCs are safer than ESCs or iPSCs in teratoma formation and gene manipulation (Sacchetti et al., 2007; Bianco et al., 2008).

Over the past two decades, studies have shown that MSCs may have potential of cell replacement due to their multilineage differentiation abilities. In addition, MSCs may play a neuroprotective role and promote neuronal repair after transplantation into the injured spinal cord via multiple biological mechanisms. For example, their paracrine activity may produce neurotrophic, immunomodulatory, and anti-inflammatory effects. However, there are still some ambiguities in this field of research, including the controversy of neural differentiation and premature clinical trials. The emergence of novel cellular and biological techniques has deepened our understanding of MSCs at the genomic, transcription, and proteome levels, which also provides new interpretations and directions in the development of MSCs treatments for SCI. As the diverse functions of MSCs are revealed, the narrative of its potential for treating SCI has been constantly updated. In this review, we discuss the development of MSCs treatments for SCI and the underlying biological mechanisms, and summarize progress and further development in this field.

## Mesenchymal Stromal Cell: An Evolving Concept

In 1968, Friedenstein discovered that a small number of bone marrow cells could adhere to Petri dishes and form fibroblast-like cells with osteogenic potential (Friedenstein et al., 1968, 1970). He also found that these cells could form colony-forming unit fibroblasts (CFU-Fs) from a single cell *in vitro* (Friedenstein et al., 1970). Subsequent studies found that these cells could differentiate into a variety of bone tissues both *in vivo* and *in vitro*, including bone, cartilage, and fat (Caplan, 1994; Horwitz et al., 2005). Thus, these multipotential cells were originally named osteogenic stem cells or bone marrow stromal stem cells (Friedenstein et al., 1987; Owen, 1988). For a long time, it was widely believed that hematopoietic stem cells were the only stem cells residing in the adult bone marrow. The discovery of these multipotential cells has brought attention to non-hematopoietic stem cells in bone marrow. Caplan et al. used the term “mesenchymal stem cell” to refer to these cells based on their multipotency to attract the attention of orthopedic research (Caplan, 1991; Pittenger et al., 1999). During this period, research in ESCs progressed rapidly, and the multipotential differentiation ability of MSCs seemed to make up the vacancy in the research of adult stem cells. In this context, many scientists considered that mesenchymal stem cells had a broader differentiation potential than expected. This hypothesis has aroused many studies investigating the trans-germ differentiation of MSCs, along with confusion and controversy (Grove et al., 2004; Lakshmipathy and Verfaillie, 2005). However, little experimental data has confirmed the stem cell properties of MSCs, and the term mesenchymal stem cells convey an assumption that deviates from the original concept of non-hematopoietic stem cells. The International Society for Cell & Gene Therapy (ISCT) suggested that these cells be named “mesenchymal stromal cells” (Horwitz et al., 2005). “Mesenchymal” indicates that the cells arise from embryonic loose connective tissue derived from mesoderm cells. “Stromal” indicates that the cells reside in the stromal/connective tissues of the supportive structures. Because the acronym “MSCs” has been widely used, retaining it avoids unnecessary confusion and maintains historical coherence. The removal of “stem” is hoped to evoke caution about its stemness and avoid exaggerating its potential. ISCT has drawn up criteria for categorizing MSCs that are widely used: MSCs (1) are plastic adherent, (2) express CD73, CD90, and CD105, but do not express hematopoietic and endothelial markers CD11b, CD14, CD19, CD34, CD45, CD79a, and HLA-DR, and (3) are capable of differentiating into adipocyte, chondrocyte, and osteoblast lineages *in vitro* (Dominici et al., 2006). Although this minimum standard has promoted research on MSCs, it has also led to the misconception that standard-compliant MSCs are identical in features and functions. In fact, later studies have demonstrated that the concept of MSCs under this framework includes different populations of cells with different biological functions.

**FIGURE 1:**
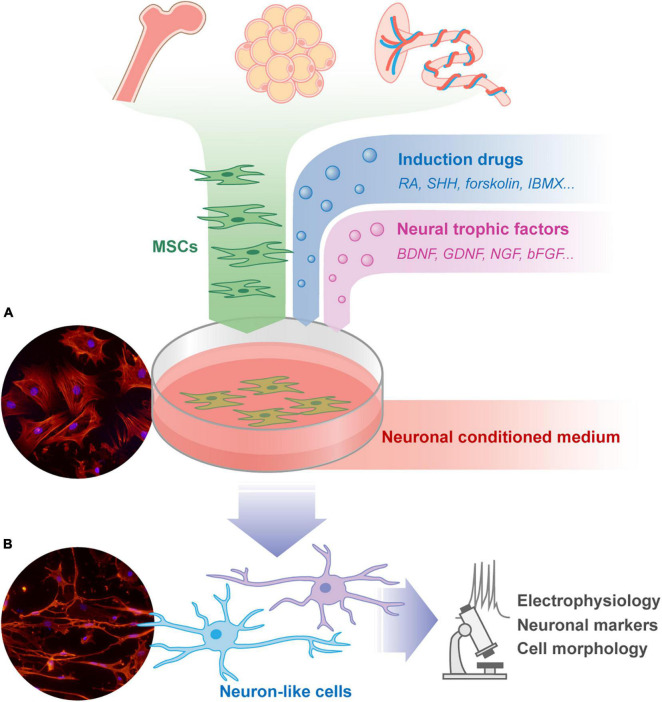
The isolation, culture, and neuronal differentiation research procedure of MSCs. MSCs can be isolated from a variety of tissues including bone marrow, fat, placenta, and so on. These cells can be cultured adherently in Petri dish, and spread out in a flat spindle shape [as shown in microscope picture **(A)**: red, Phalloidin; blue, DAPI]. The neural induction protocols of MSCs usually share some common features, including pharmacological drugs, neural trophic factors, and neuronal conditioned medium. The morphology of the differentiated MSCs will change to form neuron-like cells [as shown in microscope picture **(B)**: red, Phalloidin; blue, DAPI. Unpublished data from the author]. The trans-differentiated cells are usually verified by cell morphology, neuronal markers, and electrophysiology examination to assess the effect of treatment. MSCs, mesenchymal stromal cells; RA, retinoic acid; SHH, sonic hedgehog; IBMX, 3-isobutyl-1-methylxanthine; BDNF, brain-derived neurotrophic factor; GDNF, glial cell-derived neurotrophic factor; NGF, nerve growth factor; bFGF, basic fibroblast growth factor.

As research has progressed, the concept of MSCs has expanded from bone marrow to other tissues. MSCs have been found in almost all blood vessel-containing adult tissues, including fat (Halvorsen et al., 2000), the umbilical cord (Romanov et al., 2003), skin (Richardson et al., 2005), radices dentis (Miura et al., 2003), and menstrual blood (Bozorgmehr et al., 2020; Figure 1). The widespread distribution of MSCs may be explained by the association between its origin and blood vessels. Accumulating evidence has suggested a close association between MSCs and pericytes around vessels (Sacchetti et al., 2016). Crisan et al. (2008) demonstrated that MSCs and pericytes share a high degree of overlap in cell surface labeling, *in vivo* location, and multilineage differentiation abilities. Therefore, MSCs are hypothesized to originate from pericytes and migrate to the capillary walls of fibrous tissue after embryonic development or injury (Corselli et al., 2012; Murray et al., 2014). However, despite the similarities between pericytes and MSCs, many differences between the two cells remain, and some studies have questioned their identities (Blocki et al., 2013; Guimaraes-Camboa et al., 2017). In addition, the consistency of MSCs populations cultured *in vivo* and *in vitro* is questionable. The multipotency of MSCs may be the product of *in vitro* culture, as they retain their identity *in vivo* and do not differentiate into other lineages (Guimaraes-Camboa et al., 2017).

As the research grows, our knowledge of the biological function of MSCs in SCI treatment has undergone a paradigm shift from cell replacement to paracrine actions. Some cytokines secreted by MSCs play a major role in tissue repair and microenvironment regulation through their trophic, angiogenic, immunomodulatory, and anti-inflammatory activities (Kordelas et al., 2014). Despite the multilineage differentiation potential of MSCs, their therapeutic effects *in vivo* differ from that of conventional stem cells. In addition, MSCs do not form teratomas due to their limited differentiation capacity *in vivo*, contributing to the safety of MSCs transplantation. Based on their secretory function, Caplan (2010) suggested abandoning the notion that MSCs are stem cells and changing their name to medical signaling cells. The ISCT has also recently reiterated its position on the concept of MSCs, supporting the use of the term “mesenchymal stromal cells.” This term now describes a large unisolated heterogeneous cell population that may include fibroblasts, myoblasts, and even a small number of stem/progenitor cells, but not hematopoietic or endothelial cells (Viswanathan et al., 2019). The term “mesenchymal stem cells” should only be applied for the cell with rigorous evidence of stem cell properties both *in vitro* and *in vivo* (Viswanathan et al., 2019). The specific functions of MSCs may vary according to their source, culture method, and stimulant factors. The specific characteristics of MSCs trophic secretion, immune regulation, and vasogenic effects can be characterized using functional matrix assays, including quantitative RNA analysis of specific genes, cell marker flow cytometry, and secretory proteome analysis (Galipeau et al., 2016; Chinnadurai et al., 2018).

The evolution of the concept of MSCs shows the dynamic development in this field, and clarifying its context is helpful to deepen our understanding of MSCs. Outdated and vague understandings of concepts can and have led to obstacles to scientific research. At the same time, as a widely carried out experimental therapy in clinic, MSCs must be well-understood for both clinicians and patients to avoid misplaced expectations of its efficacy.

## Neural Differentiation Potential of Mesenchymal Stromal Cells

Due to the multipotential differentiation ability of MSCs into mesodermal cells, researchers speculated about whether MSCs could differentiate into neuronal lineages (Figure 1). MSCs are thought to develop mainly from the mesoderm (Dennis and Charbord, 2002; Wislet-Gendebien et al., 2012). The neuroectoderm produces transient populations of cells characterized as MSCs, which can persist in some adult tissues (Dupin and Coelho-Aguiar, 2013). Takashima et al. (2007) showed that MSCs in embryos are derived from SOX1^+^ neuroepithelial cells. MSCs express nestin, βIII tubulin, or neuronal characteristics in soft gels which mimic bone marrow or nerve tissue environments (Mendez-Ferrer et al., 2010; Lee et al., 2016). Transcriptome analysis of bone marrow MSCs have shown that they also express mRNA of neural tissues (Tremain et al., 2001). Genomic analyses of MSCs have shown significant overlap with master transcriptional regulators (e.g., RUNX2, C/EBPβ) that are epigenetically reduced in size after differentiation, and these promoter regions are highly plastic, leading to the trans-differentiation ability of MSCs (Wu et al., 2017). These studies serve as theoretical support for the neural differentiation potential of MSCs. Another theoretical support comes from embryological studies, which have shown that cells from one tissue can be implanted into another tissue and be locally controlled by the new environment and serve different functions.

In the last 20 years, many pioneering studies have explored the possibility neuronal differentiation of MSCs in the treatment for SCI. By transplanting MSCs into the lateral ventricles of newborn mice, Kopen et al. (1999) observed the expression of GFAP and neurofilament proteins in MSCs, suggesting that they may differentiate into nerve cells. Subsequently, MSCs cultured *in vitro* were stimulated with chemical reagents and were morphologically transformed into neuron-like cells with several different nerve cell surface markers (Sanchez-Ramos et al., 2000; Woodbury et al., 2000). Brazelton et al. (2000) observed that MSCs injected intravenously migrate to the brain and differentiate into neuron-like cells. A large number of experiments have demonstrated that there do have many culture conditions, differentiation protocols, and gene regulations that can induce MSCs to differentiate into neuron-like cells *in vitro*, express proteins that are typically expressed in nerve cells such as nerve filaments and excitatory amino acid receptors, or exhibit some electrophysiological activity (Kopen et al., 1999; Jiang et al., 2003; Dezawa et al., 2004; Hermann et al., 2004; Kondo et al., 2005; Bi et al., 2010; Ma et al., 2011; Aguilera-Castrejon et al., 2017; Kang et al., 2019). Although the culture conditions for these studies differed, they shared some common features in general, including the use of neural stem cell conditioned media, the addition of neurotrophic factors or pharmacological drugs to stimulate specific signaling pathways, and the artificial manipulation of neural cell-specific gene expressions. The pathways involved primarily included retinoic acid, Hedgehog, cAMP, Wnt, neurotrophin-activated pathways, and MAPK (Neirinckx et al., 2013; Choudhary et al., 2021). Some studies have also shown that physical methods, such as surface morphology, elasticity, and even acoustic waves, can trigger neural differentiation of MSCs (Choi et al., 2016; Mung et al., 2016; Yang et al., 2020). In addition, some adjuvant components such as butylated hydroxyanisole (BHA), β-mercaptoethanol (BME), and dimethyl sulfoxide (DMSO) are added to the medium to induce neural differentiation of MSCs, but lacking detailed explanation of their mechanisms (Woodbury et al., 2000; Kondo et al., 2005; Naghdi et al., 2009).

Although numerous studies demonstrating MSCs differentiation into neurons have been published, most are not as rigorous as the studies of the mesodermal differentiation of MSCs, and detailed experimental conditions and criteria require further identification. The reprograming of stem cell phenotypes depends on the appropriate cellular environment and sustained application of instructive agents. The developmental biology of MSCs in the embryonic stage is far different from that of adult tissue repair and induction culture environments. Most existing studies assessed the neuronal induction of MSCs via cell morphology and the expression of classical neural markers, and some have examined ion channel properties (Tropel et al., 2006; Neirinckx et al., 2013). However, the criteria of cell differentiation requires further clarification: differentiation into neurons does not simply mean that the cell has a similar morphology or immune-phenotype to that of a nerve cell, but more importantly, that the differentiated cell has the ability to receive and transmit neural signals, including releasing neurotransmitters or activating action potentials. Morphological changes and neural marker expression of trans-differentiated MSCs may be artifacts, and electrophysiological activity cannot be equated with action potential triggering and conduction (Montzka et al., 2009). In fact, it has been suggested that although trans-differentiated MSCs have a neuronal phenotype, they lack functional action potentials (Barnabe et al., 2009). Lu et al. (2004) summarized several studies of the drug-induced differentiation of MSCs into nerve cells. Although cytoplasmic wrinkling and increased expression of neural markers were observed in different cell types treated with these drugs, these phenomena were also observed when MSCs were treated with cytotoxic agents. Therefore, Lu et al. (2004) suggested that neural differentiation of MSCs may be morphological changes and neural marker expression artifacts caused by cytoplasmic shrinkage, which was itself triggered by cellular stress responses. Neuhuber et al. (2004) reported similar observations. Neural markers are a necessary but not sufficient condition for neural differentiation of MSCs. The neural ectodermal origin of some MSCs may explain the early results showing that MSCs share some surface markers with neural lineage cells (Phinney and Prockop, 2007). For example, nestin, a classic marker of neural stem cells, is also expressed in subtypes of MSCs that are capable of osteogenesis and angiogenesis (Mendez-Ferrer et al., 2010; Baryawno et al., 2019). In addition, transcriptome analysis of MSCs after induction has shown that the increased expression of nestin may be due to the upregulation of cytoskeleton-related proteins; that is, it may be based on morphological changes rather than changes in cell function (Khan et al., 2020).

In conclusion, current research indicates that the evidence for neural differentiation of MSCs is not strong enough, and it remains difficult to differentiate MSCs into functional mature nerve cells *in vitro* using current culture regimens. Therefore, a more cautious attitude is needed to review studies of neural differentiation of MSCs in multiple dimensions.

## Mesenchymal Stromal Cells and Nerve Regeneration

**FIGURE 2:**
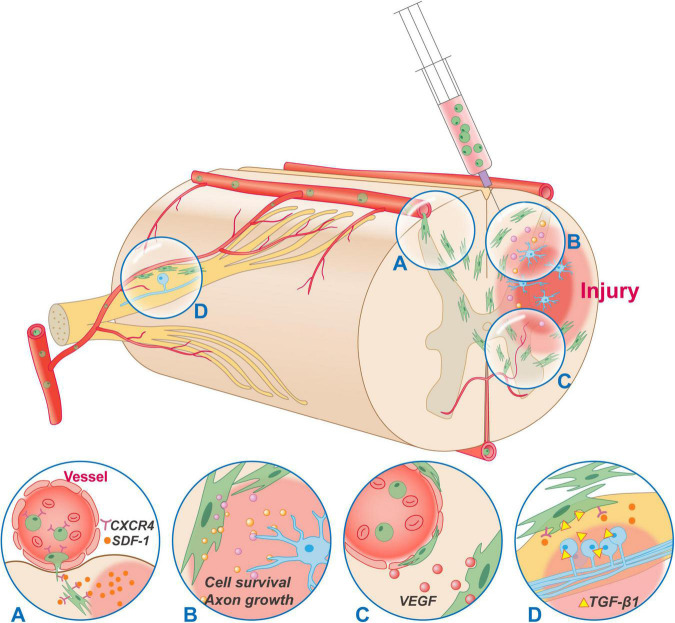
Typical mechanisms of MSCs transplantation in the treatment of SCI. MSCs can be administrated by intravenous, intraspinal, and subarachnoid injection. Regardless of the administration route, MSCs can migrate and accumulate to the injury site. **(A)** The migration of MSCs is conducted through the SDF-1/CXCR4 axis. **(B)** MSCs secrete a variety of neurotrophic and immunoregulatory factors through a bystander effect to regulate the microenvironment of injury site, rescue cell death and promote axon growth. **(C)** Most MSCs reside around the vessels and have some functionalities in common with those of pericytes. Besides, MSCs secrete VEGF constitutively without being regulated by inflammatory factors, which has a positive effect on improving vascular injury, ischemia, hypoxia, and the accumulation of inflammatory substances after SCI. **(D)** MSCs can migrate to the DRG through SDF-1/CXCR4, and secrete anti-inflammatory factors such as TGF-β1, IL-10, LIF-10 to inhibit hyperexcitability of DRG cells, alleviate opioid tolerance, and relieve hyperalgesia. CXCR4, CXC chemokine receptor type 4; SDF-1, stromal cell-derived factor-1; VEGF, vascular endothelial growth factor; TGF-β1, transforming growth factor beta 1; IL-10, interleukin-10; LIF-10, leukemia inhibitory factor.

Although the neural differentiation and cell replacement functions of MSCs are controversial, the beneficial effects of MSCs on SCI have indeed been observed by researchers. These benefits may not result from the cellular replacement of MSCs, but rather from the paracrine and immune regulatory roles of MSCs. Contrary to the belief that cells behave the same way in tissue culture as they do *in vivo*, MSCs did not differentiate into cartilage, bone, or fat *in vivo* (Caplan, 2017). Zwolanek et al. (2017) found that MSCs did not differentiate directly into cartilage during cartilage repair as expected, but rather acted through non-progenitor cell pathways. A similar situation occurred during nerve damage repair. One possible explanation is that due to the role of MSCs themselves in supporting and maintaining the hematopoietic microenvironment and the blood sinus network in bone marrow, the transplantation of MSCs may lead to the transfer of their inherent biological functions to target organs, namely, non-hematopoietic cells may obtain the nursing effect from direct or paracrine interaction with MSCs (Caplan and Dennis, 2006; Bianco et al., 2008). Gene expression analysis of MSCs has shown that MSCs are not only involved in hematopoietic support but are also involved in angiogenic, anti-inflammatory, and immunomodulatory activities (Tremain et al., 2001; Pittenger et al., 2019). Many studies have found that growth factors, cytokines, and other bioactive substances produced by MSCs are contained in exosomes and microvesicles, which play a paracrine role in stimulating tissue repair, regulating inflammation, modulating immunity, promoting angiogenesis, and repairing nerve injury (Table 1; Baglio et al., 2015; Phinney and Pittenger, 2017). These secretory effects change dynamically with the microenvironment *in vivo* or at the site of injury, and in turn, affect the local microenvironment. This “plasticity” and “crosstalk” are the key to the therapeutic effects of MSCs (Phinney and Sensebe, 2013). At the same time, this also makes exploration of the therapeutic effects of MSCs more complicated, and it is difficult to identify the specific role of certain cell factors in neuroprotection. The comprehensive role of these pathways is not fully understood because of the synergies and overlap of these mechanisms in function, and how this process dynamically responds to SCI damage environmental cues, cell cloning, and culture environment has not been fully revealed. Therefore, reviewing the secretory characteristics of MSCs in SCI is the key to understanding their biological functions (Figure 2).

**TABLE 1:** Mesenchymal stromal cells (MSCs) secrete a variety of growth factors, cytokines, and bioactive substances to take effect in spinal cord injury.

Factors	Function in SCI repairment	References
bFGF	Stimulate the proliferation and migrations of NSCs, promote neuron regeneration and anti-inflammation.	Woodbury and Ikezu, 2014
VEGF	Promote angiogenesis in spinal cord.	Okon et al., 2013; Teixeira et al., 2017
BDNF	Neuroprotection, promote the growth and differentiation of neurons and synapses.	Teixeira et al., 2017
NGF	Neurotrophic function, promote the growth, maintenance, proliferation, and survival of neurons, promote the survival of sympathetic and sensory neurons.	Ribeiro et al., 2012; Kolar et al., 2017
GDNF	Neuroprotection, support the survival of dopaminergic and motor neurons, reduce apoptosis of motor neurons, reduce axotomy-induced cell death.	Hoban et al., 2015
IL-6	Attract phagocytic cells, scavenging superoxide radicals by increasing the antioxidant enzyme activity.	Kilroy et al., 2007; Lindsay et al., 2016
IL-10	Relieve hyperalgesia of DRG neurons.	Li et al., 2018
SDF-1	Regulate cell migration, recruit the NSCs and MSCs to the injury site, promote axon growth and neurogenesis by providing guiding for axons and neurites.	Li et al., 2012; Bang et al., 2017
TNF-α	Attracting phagocytic cells; promote polarization of T cells to Th1 phenotype to increase cell-mediated immune reaction.	Bernardo and Fibbe, 2013; Jin et al., 2016
GDN	Neuroprotection, promote neurite outgrowth through prevention of oxidative stress.	Hoffmann et al., 1992; Teixeira et al., 2017
PEDF	Neurotrophic function, induce the expression of BDNF and GDNF, reduce oxidant-induced neuronal death, promote axon regeneration.	Falk et al., 2010; Sanchez et al., 2012
TGF-β	Promote the growth of neurites, induce formation of axons; promote migration of immature neurons at low concentration, impair migration at high concentration; inhibit hyperexcitability of DRG neurons, relieve hyperalgesia.	Unsicker et al., 1996; Yi et al., 2010; Chen et al., 2015
TIMP-1	Promote oligodendrocyte differentiation of NSCs, promote formation of myelin sheath.	Samper Agrelo et al., 2020
IDO1	Reduce inflammation by consuming tryptophan, but increase cell death under oxygen and glucose deprivation.	Krampera et al., 2006; Wang et al., 2020
TSG-6	Suppressed the inflammation cascade inducted by NF-κB signaling pathway in resident macrophages; promote macrophage from pro-inflammatory to anti-inflammatory phenotype.	Choi et al., 2011; Mittal et al., 2016

*bFGF, basic fibroblast growth factor; VEGF, vascular endothelial growth factor; BDNF, brain-derived neurotrophic factor; NGF, nerve growth factor; GDNF, glial cell-derived neurotrophic factor; IL-6, interleukin-6; IL-10, interleukin-10; SDF-1, stromal cell-derived factor-1; TNF-α, tumor necrosis factor; GDN, glia-derived nexin; PEDF, pigment epithelium-derived factor; TGF-β, transforming growth factor β; TIMP-1, tissue inhibitor metalloproteinase type-1; IDO1, indoleamine 2, 3-dioxygenase; TSG-6, TNF-α-stimulated gene 6 protein.*

### Migration Ability of Mesenchymal Stromal Cells

The migration, or homing, of MSCs is their ability to migrate to injured tissues/organs. This migration concentrates the biological function of MSCs at the site of SCI and is the basis for the feasibility of intravenous or intrathecal injection of MSCs. MSCs migration is regulated by a variety of factors, and the key chemokine is stromal cell-derived factor-1 (SDF-1, also known as CXCL12) (Liu et al., 2011). SDF-1 levels can be locally elevated due to pathological conditions such as inflammation, ischemia, and hypoxia (Li and Ransohoff, 2008). SDF-1 binds to CXC chemokine receptor 4 (CXCR4) on the surfaces of MSCs to activate signaling molecules, including PI3K, ERK, and Akt, which attract MSCs to the site of injury (Bang et al., 2017). In addition, substance P released after SCI impairs transforming growth factor β (TGF-β) mediated MSCs mobilization but does not affect the function of SDF-1 (Nam et al., 2020). A variety of growth factors, including basic fibroblast growth factor (bFGF), hepatocyte growth factor (HGF), leukemia inhibitory factor (LIF), vascular endothelial growth factor (VEGF), IGF-1, and VE-cadherin, also participate in the migration of MSCs (Karp and Leng Teo, 2009; Nitzsche et al., 2017; Fu et al., 2019).

### Trophic Function of Mesenchymal Stromal Cells

A widely proposed mechanism by which MSCs promote SCI recovery is the trophic factors or cytokines secreted by MSCs, which play an important role in regulating endogenous repair after SCI in many aspects. Chopp and Li (2002) demonstrated the presence of important neuroregulatory molecules in the secretome of MSCs, including vascular endothelial growth factor (VEGF), nerve growth factor (NGF), brain-derived neurotrophic factor (BDNF), basic fibroblast growth factor (bFGF), insulin-like growth factor-1 (IGF-1), and glial cell derived neurotrophic factor (GDNF), which play an important role in neurogenesis and angiogenesis. Furthermore, Teixeira et al. (2017) analyzed untreated bone marrow MSCs and observed secretion of other factors, such as interleukin-6 (IL-6), cystatin C, glia-derived nexin (GDN), galectin-1, and pigment epithelium-derived factor (PEDF), which plays an important role in cell migration, differentiation, and neuroprotection. BDNF is believed to have the ability to induce neuronal differentiation and axon growth (Fujii et al., 2015; Ritfeld et al., 2015), and its protective effect is mediated partly by activation of the AKT pathway (Wilkins et al., 2009). Direct transplantation of BDNF-overexpressing bone marrow MSCs to treat nerve injury increases nerve tissue volume and vascular density (Ritfeld et al., 2015). Gunther et al. (2015) used an alginate scaffold to carry BDNF-modified MSCs and observed a large number of axons regenerated and penetrated across the injury site. However, Brock et al. (2016) transplanted MSCs genetically modified to secrete BDNF into spinal cord contusion injuries in rats and observed no reduction in the size of the damaged area or improvement in motor function. GDNF plays an important role in the improvement of motor function and has antioxidant effects, enhancing the activity of enzymes involved in detoxification of reactive oxygen species (e.g., superoxide dismutase, catalase, and glutathione) (Hoban et al., 2015). Up-regulation of PEDF stimulates the cascade activation of the NF-kB signal, induces the expression of BDNF and GDNF, and triggers improvement in behavior and survival of neurons (Falk et al., 2010). In addition, Sanchez et al. (2012) found that PEDF can reduce the oxidant-induced neuronal death through reducing phosphorylation of extracellular signal-regulated kinase 1/2. Samper Agrelo et al. (2020) analyzed the secretome of bone marrow MSCs transplanted into the nervous system and found increased survival of neural stem cells stimulated by transplanted MSCs. Moreover, the tissue inhibitor metalloproteinase type 1 (TIMP-1) secreted by MSCs promoted the differentiation of NSCs into oligodendrocytes, which promoted the formation of regenerative myelin sheaths (Samper Agrelo et al., 2020). MSCs can also secrete VEGF constitutively without being regulated by inflammatory factors, which has a positive effect on improving vascular injury, ischemia, hypoxia, and the accumulation of inflammatory substances after SCI, thereby promoting the repair of nerve tissues (Okon et al., 2013; Teixeira et al., 2017). In addition, it was found that hypoxia (O_2_ < 5%) can induce MSCs to enhance nerve growth and angiogenesis capacity (Lennon et al., 2001; Rosova et al., 2008; Tsai et al., 2011; Liu et al., 2013). The up-regulated expression of growth factors such as BDNF, GDNF, IGF-1, and bFGF after MSCs transplantation can also promote the formation of immature blood vessels (Li et al., 2021).

### Immune Regulation of Mesenchymal Stromal Cells

Multiple factors secreted by MSCs are involved in local inflammatory responses following SCI. Chinnadurai et al. (2018) analyzed the immunomodulatory function of the secretome of MSCs using a combinatorial assay matrix method and found that it involves altering the active regulatory pathway in immune response, which plays a major role in the immune regulation of host tissues. MSCs can also exert anti-inflammatory or immune regulation effects by responding to inflammatory stimuli. For example, exposure to interferon-gamma (IFN-γ) causes MSCs to bring forth immunosuppressive activity by expressing indoleamine 2, 3-dioxygenase (IDO1), an enzyme that consumes tryptophan to reduce inflammation (Krampera et al., 2006). In addition, Galleu et al. (2017) reported that the apoptosis of MSCs induced by recipient cytotoxic cells after transplantation is essential to initiate immunosuppression. Apoptotic MSCs are engulfed by phagocytes and induce the latter to produce IDO1. The MSCs also secrete the TNF-α-stimulated gene 6 protein (TSG-6) when activated by inflammatory signals and suppress the inflammation cascade induced by NF-κB signaling pathway in resident macrophages, and shift macrophages from pro-inflammatory to anti-inflammatory phenotype (Choi et al., 2011; Mittal et al., 2016). In addition, IFN-γ and tumor necrosis factor (TNF-α) promoted the polarization of MSCs toward a secretory phenotype characterized by the expression of IL-4, IL-10, CD274, PD-L1, and IDO (Jin et al., 2016). Besides, the transplanted MSCs may also be phagocytosed by monocytes and induce the latter shift to immunomodulatory intermediate phenotype with anti-inflammatory abilities (Bernardo and Fibbe, 2013; de Witte et al., 2018). However, exposure to inflammatory stimuli also has adverse effects on MSCs. For example, IFN-γ can induce programed cell death of MSCs (Bernardo and Fibbe, 2013). In addition, procedures in cell transplantation, such as freezing and thawing, result in a defective secretome of MSCs and reduce their survival rate in inflammatory environments (Pollock et al., 2015; Chinnadurai et al., 2016).

### Relief of Neuropathic Pain by Mesenchymal Stromal Cells

Neuropathic pain is a common complication following SCI and often seriously affects the physical and mental state of patients. Opioids are a common drug to treat neuropathic pain but often cause opioid tolerance and induce hyperalgesia. MSCs transplantation has shown favorable analgesic effects in these patients. Chen et al. (2015) found that bone marrow MSCs injected into the subarachnoid space of the spinal cord recruited to the dorsal root ganglion (DRG) through SDF-1/CXCR4, and secreted TGF-β1 to inhibit hyperexcitability of DRG cells and relieve hyperalgesia. Both intrathecal and intravenous injection of MSCs can alleviate opioid tolerance and opioid-induced hyperalgesia, the mechanisms of which may be related to TGF, IL-10, and LIF-10 secreted by MSCs (Li et al., 2018). Hua et al. (2016) reported similar results. Vaquero et al. (2018a) reported that MSCs transplantation significantly improved neuropathic pain in patients in a clinical trial. In conclusion, the relief of neuropathic pain conducted by MSCs transplantation brings additional benefits for SCI treatment.

### Nestin^+^ Mesenchymal Stromal Cells in Neural Repair

Nestin, a type VI intermediate filament protein, is regarded as a neural stem cell marker, as well as expressed in fibroblasts, endothelial progenitor cells, and some bone marrow MSCs (Lendahl et al., 1990; Mokry et al., 2004; Xie et al., 2015). Nestin^+^ MSCs co-localize with hematopoietic stem cells (HSCs) and constitute an important component of the HSCs niche (Mendez-Ferrer et al., 2010). The Nestin^+^ group of MSCs has some potential superior characteristics that may make it a candidate for the treatment of SCI. Nestin^+^ MSCs have stronger self-renewal and multilineage differentiation potential than Nestin^–^ MSCs and express higher levels of chemokine SDF-1, stem cell factor, angiopoietin-1, and vascular cell adhesion molecule 1 (VCAM1) (Lindsay and Barnett, 2017; Lu et al., 2019). The secretion of SDF-1 by Nestin^+^ MSCs is regulated by the sympathetic nerve, and the SDF-1 not only promotes the recruitment of NSCs to the injury site but also provides guiding cues for axons and neurites, thus promoting axon growth and neurogenesis (Li et al., 2012; Guyon, 2014). Lindsay et al. (2013) found that Nestin^+^ MSCs isolated from olfactory mucosa could better promote the myelination of the central nervous system *in vitro*. In addition, Nestin^+^ MSCs show strong potential for angiogenesis (Pacini and Petrini, 2014). Due to these unique biological characteristics of Nestin^+^ MSCs, they may be a good candidate for repairing SCI, or one of the entry points for us to understand the biological function of MSCs fully.

## Challenges

### Improving Cell Transplantation Techniques

The efficacy of MSCs transplantation is affected by the time and route of administration, as well as the number of cells. Administration routes include intravenous, intraspinal, and subarachnoid injection. Many preclinical and clinical studies have reported on the safety of MSCs transplantation by these routes, but there is no sufficient evidence to show that one route is superior to others (Table 2; Zhao et al., 2019; Zholudeva and Lane, 2019). The intravenous injection is less invasive, but there is a risk of cells sticking together that forms microemboli and block the vascular system. In addition, a large proportion of cells are trapped in peripheral organs and circulation due to the blood-spinal barrier. Direct intraspinal injection of MSCs into the injured area of the spinal cord can achieve a high number of cells in the site of transplantation. However, the harsh microenvironment of the injured spinal cord contains inflammatory factors and ion disturbances, which affect the survival and proliferation of transplanted cells. In addition, the volume effect of injecting a large number of cells may cause secondary damage. Since MSCs function through bystander effect rather than cell replacement, transplanting them around the injury site instead right in the epicenter can make them take effect while avoiding the adverse effects of the harsh microenvironment and improving the survival rate of cells. Current clinical studies have shown no significant difference in the efficacy of intravenous or intrathecal injection of MSCs, which may be due to the migration effect of MSCs (Muthu et al., 2021).

**TABLE 2:** Main clinical studies on mesenchymal stromal cells (MSCs) therapy for spinal cord injury (SCI) in recent 10 years.

Studies	MSCs source	Cell count	Method of transplant	Outcomes	Reported adverse events
Yang et al. (2021)	Allogenic UC	1 × 10^6^ cells/kg, four doses	Subarachnoid	Improvements in pinprick, light touch, motor, sphincter, bladder and bowel functions. Decrease in muscle spasticity.	Fever, headache, dizziness, nausea.
Oraee-Yazdani et al. (2021)	Autologous BM and Schwann cells	5 × 10^7^	Intrathecal	Improvements in trunk movement, body stability, bladder and rectal sensation; reduction in constipation.	Mild headache, neuropathic pain, numbness spasticity.
Vaquero et al. (2018b)	Autologous BM	3 × 10^8^	Intra-spinal cord	Improvements in sensation, neuropathic pain, bowel and bladder function, voluntary movements.	Bronchopneumonia in one patient.
Vaquero et al. (2017)	Autologous BM	3 × 10^7^, four doses	Subarachnoid	Improvements in motor and sensory function; reduction in neuropathic pain.	Headache, pain in puncture site.
Larocca et al. (2017)	Autologous BM	2 × 10^7^	Intra-spinal cord	Improvements in bowel movements and regularity, recovery in sensation.	No adverse event.
Satti et al. (2016)	Autologous BM	1.2 × 10^6^ cells/kg	Intrathecal	Not evaluated.	No adverse event.
Oh et al. (2016)	Autologous BM	1.6 × 10^7^ + 3.2 × 10^7^	Intra-spinal cord, Subdural	Improvements in neurological status (2 out of 12 patients).	No adverse event.
Hur et al. (2016)	Autologous AD	9 × 10^7^	Intrathecal	Improvements in motor and sensory function, anal contraction.	Urinary tract infection, headache, nausea, vomiting.
Mendonca et al. (2014)	Autologous BM	5 × 10^6^ cells/cm^3^	Intra-spinal cord	Improvements in lower limbs motor function, urologic function.	Cerebrospinal fluid leakage.
El-Kheir et al. (2014)	Autologous BM	1.2 × 10^6^ cells/kg	Intrathecal	46% patients got improvements in functional measurements.	No adverse event.
Cheng et al. (2014)	Allogenic UC	2 × 10^7^, two doses	Intra-spinal cord	Improvements in motor, urologic functions and muscular tension.	One patient got neuralgia within 24 h after surgery.
Jiang et al. (2013)	Autologous BM	1 × 10^8^	Intra-spinal cord	Improvements in motor, sensory and autonomic nerve functions.	Fever, headache within 24–48 h after surgery.
Dai et al. (2013)	Autologous BM	1 × 10^8^	Intra-spinal cord	Improvements in motor, sensory function and residue urine volume.	Fever, headache, dizziness.

*AD, adipose-derived; BM, bone marrow; MSCs, mesenchymal stromal cells; SCI, spinal cord injury; UC, umbilical cord.*

The timing of cell transplantation is also a key issue to consider because the changes in the local environment over time after injury will affect the survival and proliferation of the transplanted cells, as well as the mechanism by which they take effect. SCI can be temporally divided into acute (<48 h), subacute (48 h to 14 days), intermediate (14 days to 6 months), and chronic phases (>6 months) (Ahuja et al., 2017b). The major problem of cell transplantation during the acute phase is that the pro-inflammatory environment at the lesion site adversely affects the cell survival, but in turn, the surviving cells can secrete anti-inflammatory or neuroprotective factors to take effect in the early stage. The subacute phase is characterized by continuous anatomical and biochemical changes in the spinal cord, during which cell transplantation may take effect in trophic support and angiogenesis, with a relatively better survival rate (Lin et al., 2017). Four weeks after the SCI, the anatomy and physiology condition of the injured spinal cord become stable, and glial scars are formed as a barrier to nerve regeneration. It is unclear whether the paracrine ability of MSCs can affect the chronic glial scars, and whether the benefits of transplantation during this stage are comparable to those of earlier treatments. However, the research on cell transplantation in the chronic phase is of great significance, as most SCI patients are living in this period, and more research should focus on this area.

The dose of cell transplantation varies widely, ranging from 1 × 10^5^ to 1 × 10^9^ (Andrzejewska et al., 2021). A meta-analysis of clinical studies on MSCs transplantation for SCI showed that therapeutic effects occurred with a low dose of MSCs (<5 × 10^7^ cells) injected intravenously or intrathecally (Muthu et al., 2021). Another meta-analysis showed that transplant dose 1–5 × 10^7^ between 10–20 × 10^7^ may provide more benefits for patients with SCIs (Zhao et al., 2019). However, Krupa et al. (2018) showed a dose-dependent effect of intrathecal injection of MSCs in the treatment of SCI, and multiple and high-dose cell transplants (1.5 million × 3 times) have better efficacy. Similar results have been reported in studies by Vaquero et al. (2016). It is difficult to identify the ideal dose of transplanted cells, but a large dose does not seem to affect the safety of MSCs transplantation.

### Remaining Ethical and Scientific Dilemmas

The ethical rationality of MSCs transplantation is based more on the observed safety than the in-depth understanding of its mechanisms. The ISCT minimal standards for MSCs have promoted research on this field, but relying on these standards alone has led to the misconception that standards-compliant MSCs are identical in characteristics and functions. In fact, MSCs are not merely a collection of surface markers but have huge heterogeneity due to differences in cell sources, culture methods, and stimulation methods, and the biological features of MSCs *in vivo* and *in vitro* are not exactly the same (Le Blanc and Davies, 2018; Pittenger et al., 2019). In addition, while MSCs play a complex role *in vivo* and affect the local environment, they can also be influenced by the host tissues, which increases the complexity of their function. These scientific uncertainties bring about ethical flaws, since in the absence of thoroughly clarifying conditions and mechanisms, the clinical application of MSCs in the treatment of SCI lacks a steady scientific foundation. However, from a practical point of view, the research history of cell transplantation and an abundance of clinical trial data do show that there are no significant adverse reactions to MSCs in the treatment of SCI, no matter what kind of administration route. Given the plight of SCI patients, such a treatment may be worth taking a try, but it must be performed with strict supervision and full informed consent.

Given the heterogeneity of MSCs and their susceptibility in culture conditions *in vitro*, researchers must recognize the fact that different culture conditions will produce different MSCs products, even if they meet the minimum standards of MSCs. There are dozens of clinical trials of MSCs for the treatment of SCI on the registry; however, the heterogeneity of the MSCs in these studies reduces the comparability of data (Pittenger et al., 2019). To address this issue, bioequivalence standards have been derived from the iPSC library to create a stable, differentiable benchmark against which MSCs products can be compared (Viswanathan et al., 2014; Prockop, 2017). The study of MSCs therapy requires the incorporation of technical systems for the isolating, culturing, and purifying of MSCs from different sources into research, and carrying out standardized testing of *in vitro* genetic stability and efficacy testing of disease-specific mechanisms (Yin et al., 2019). Standardizing the quality and characteristics of MSCs products using these methods would improve the comparability of studies and reduce ethical issues.

## Summary

We reviewed the conceptual evolution of MSCs and summarized the mechanisms of MSCs in treating SCI. The ease of acquisition and expansion of MSCs, as well as their abundant trophic functions, anti-inflammatory, and immunomodulatory activities, make them an attractive cellular tool for the treatment of SCI. However, the complexity of the identity and function of MSCs is the other side of the coin, and we should admit that our understanding of MSCs is incomplete. Our understanding of MSCs and its role in the treatment of SCI updates with the development of biological methods. As a next step, efforts to trans-differentiate MSCs into neural lineages should be incorporated into a more rigorous framework. Another goal is to identify the specific mechanisms of MSCs with different characteristics in SCI and how they respond to the changing environments of injury. Clarifying these issues is essential for the final clinical application of MSCs therapy.

## Author Contributions

J-LX illustrated the figures. All authors provided input, co-wrote the manuscript, and approved the submitted version.

## Conflict of Interest

The authors declare that the research was conducted in the absence of any commercial or financial relationships that could be construed as a potential conflict of interest.

## Publisher’s Note

All claims expressed in this article are solely those of the authors and do not necessarily represent those of their affiliated organizations, or those of the publisher, the editors and the reviewers. Any product that may be evaluated in this article, or claim that may be made by its manufacturer, is not guaranteed or endorsed by the publisher.
